# Practical and Compact Guided Mode Resonance Sensing System for Highly Sensitive Real-Time Detection

**DOI:** 10.3390/s25134019

**Published:** 2025-06-27

**Authors:** Yen-Song Chen, Devesh Barshilia, Chia-Jui Hsieh, Hsun-Yuan Li, Wen-Hsin Hsieh, Guo-En Chang

**Affiliations:** Department of Mechanical Engineering, and Advanced Institute of Manufacturing with High-Tech Innovations (AIM-HI), National Chung Cheng University, Chiayi 62102, Taiwan; rtdfgcvb22@gmail.com (Y.-S.C.); carlo860828@gmail.com (C.-J.H.); leonli308@outlook.com (H.-Y.L.); imewhh@ccu.edu.tw (W.-H.H.)

**Keywords:** real-time detection, guided mode resonance, optical detection system, Jones matrix, refractive index sensing

## Abstract

Guided mode resonance (GMR) sensors are known for their ultrasensitive and label-free detection, achieved by assessing refractive index (RI) variations on grating surfaces. However, conventional systems often require manual adjustments, which limits their practical applicability. Therefore, this study enhances the practicality of GMR sensors by introducing an optimized detection system based on the Jones matrix method. In addition, finite element method simulations were performed to optimize the GMR sensor structure parameter. The GMR sensor chip consists of three main components: a cyclic olefin copolymer (COC) substrate with a one-dimensional grating structure of a period of ~295 nm, a height of ~100 nm, and a ~130 nm thick TiO_2_ waveguide layer that enhances the light confinement; an integrated COC microfluidic module featuring a microchannel; and flexible tubes for efficient sample handling. A GMR sensor in conjunction with a specially designed system was used to perform RI measurements across varying concentrations of sucrose. The results demonstrate its exceptional performance, with a normalized sensitivity (*S*_n_) and RI resolution (*R*_s_) of 0.4 RIU^−1^ and 8.15 × 10^−5^ RIU, respectively. The proposed detection system not only offers improved user-friendliness and cost efficiency but also delivers an enhanced performance, making it ideal for scientific and industrial applications, including biosensing and optical metrology, where precise polarization control is crucial.

## 1. Introduction

Optofluidic-based biosensing systems have emerged as a cutting-edge technology with the potential to revolutionize biosensing capabilities for a wide domain of applications, including food safety, environmental monitoring, biomedical detection, and chemical analysis [1,2,3,4]. By integrating optical sensing mechanisms with microfluidic sample handling, optofluidic biosensors provide a miniaturized, robust, and highly sensitive platform for detecting biomolecular interactions in real time. These systems typically comprise two core components: an optofluidic optical sensor that serves as a transducer, converting changes in the analyte concentration into optical signals, and a readout system that captures these optical signals, facilitating a sensitive label-free detection.

Various biosensing systems based on optofluidic technology have been demonstrated for biodetection, including prisms [5,6], interferometers [7,8,9], surface plasmon resonance (SPR) [10,11,12,13,14,15,16], fiber-optic biosensors [17,18,19,20,21], photonic crystal (PhC) [22,23], optical resonators [23,24,25], waveguides [26,27,28], and 1D gratings [2,29]. The three-layer terahertz four-band absorber based on Dirac semimetals demonstrates an exceptional refractive index (RI) sensitivity of 1840 GHz/RIU, highlighting its potential for high-performance sensing applications [30]. The inverse T-shaped structure, composed of vertically and horizontally coupled cavities integrated with a truncated bus waveguide, demonstrates a promising performance for sensing applications, particularly in CO_2_ detection [31]. Among these technologies, guided mode resonance (GMR) sensors that utilize refractometry to monitor variations in the grating surface RI for distinct concentration analytes have attracted considerable interest, owing to their straightforward device configuration, exceptional integration capabilities, and enhanced sensitivity [29,32]. Additionally, the sharp resonance features associated with GMR enable ultra-narrow linewidths and a strong optical confinement, which in turn enhance the signal-to-noise ratio and detection resolution. These features collectively position GMR biosensors as highly favorable candidates for integration into next-generation biosensing platforms, particularly those requiring real-time, high-throughput, and multiplexed detection, such as portable point-of-care diagnostic devices and lab-on-chip systems. These advantages make GMR biosensors highly promising for integration into real-time, multiplexed point-of-care diagnostic platforms.

GMR sensors use periodic structures such as gratings to support leaky guided modes that generate an evanescent field along the sensor surface. When incident light meets the resonance condition, it couples into the waveguide layer, producing a sharp resonance at a specific wavelength or angle. This resonance is highly sensitive to changes in the surrounding RI, which makes GMR sensors ideal for detecting the binding of target biomolecules to the sensor surface. As molecules interact with the sensing region, the local RI changes, resulting in a measurable shift in the resonance signal. This label-free sensing approach allows for a high sensitivity and real-time detection, which has been widely explored for applications in chemical, biological, and environmental analysis [2,29,30,31,32,33,34,35,36,37,38,39]. Despite their strong performance, the practical implementation of GMR-based optofluidic sensing systems still faces two major challenges. First, most high-sensitivity GMR sensors rely on complex and expensive instrumentation, such as tunable light sources, high-resolution spectrometers, or precise angular scanning setups to accurately detect minute shifts in the resonance condition. Second, these systems often require extensive and time-consuming data processing, which limits their speed and efficiency [2,29]. To overcome these issues, researchers have been actively working on simplifying the detection method by moving away from wavelength or angle-resolved measurements and instead focusing on intensity-based detection [2,34]. This approach significantly reduces equipment costs and simplifies the overall system design. However, intensity-based systems have shown a lower detection performance, especially in terms of the limit of detection (LOD), which limits their ability to identify low-concentration targets with a high precision. Enhancing the LOD of such systems is therefore a key step toward making GMR-based sensors more practical for real-world use. One promising strategy to improve both sensitivity and usability involves leveraging the properties of polarized light. Polarization-based techniques offer deeper insight into how light interacts with biological samples and can be used to monitor subtle changes in the optical signal with high fidelity.

In this vein, the Jones matrix a mathematical framework for describing the behavior of polarized light provides a powerful tool for characterizing changes in the polarization state as light passes through different media. By integrating the Jones matrix analysis into the GMR sensing system, the need for manual optical adjustments is eliminated, simplifying the experimental setup. This not only reduces the alignment complexity and improves reproducibility, but also allows for real-time measurements, bringing GMR-based biosensing systems closer to practical deployment in portable, cost-effective, and user-friendly diagnostic devices.

In this study, we developed a sensitive GMR sensor in conjunction with a specially designed low-cost optical detection system for real-time detection, aiming to address the limitations of conventional GMR systems. Previous setups often required skilled manual adjustments of incident angles, which hindered their practicality. Although an intensity detection-based GMR sensor is not a novel concept in optical sensing, the novelty of the proposed work lies in the integration of a Jones matrix-based detection approach specifically tailored for GMR sensors. The Jones matrix formalism is a convenient mathematical tool to describe the polarization behavior of the detection system components; however, it is not central to the detection mechanism. The proposed method enables the real-time control of the polarization state without the need for manual adjustments during RI measurements. Consequently, the overall system complexity is significantly reduced, and the operational efficiency is improved. By eliminating the dependence on manual polarization tuning, the proposed system enhances usability and reliability. Furthermore, the detection system resolves challenges associated with overlapping signal light sources and optical signals, thereby enhancing the measurement accuracy and usability. In addition to the optical detection system design, simulations were conducted to optimize the GMR sensor chip parameters, achieving an optimized resonance wavelength ($\lambda_{R})$ closer to the resonance wavelength of the green LED light source. The proposed GMR sensing system can detect RI changes with the system resolution of $R_{s}=8.15\times 10^{-5}\;\mathrm{RIU}$ within 5 min, positioning it as a promising tool for advancing biosensing technologies. Additionally, the designed optical detection system reduces the operational complexity and cost by eliminating the requirement for spectrometers to validate resonant wavelength positions. This feature makes the system user-friendly and operatable even by non-expert operators, highlighting the novelty of this study. With its rapid response time, simplicity, and high sensitivity, this system holds significant potential to revolutionize disease diagnosis and patient care in the near future.

The rest of this paper is organized as follows: Section 2 presents the design and sensing principle. Section 3 describes the materials and methods, including the fabrication process and the design of the optical detection system for the proposed sensor. Section 4 summarizes the simulation and RI sensing results, and Section 5 provides the conclusions.

## 2. Design and Sensing Principle of GMR Sensor

**Figure 1:**
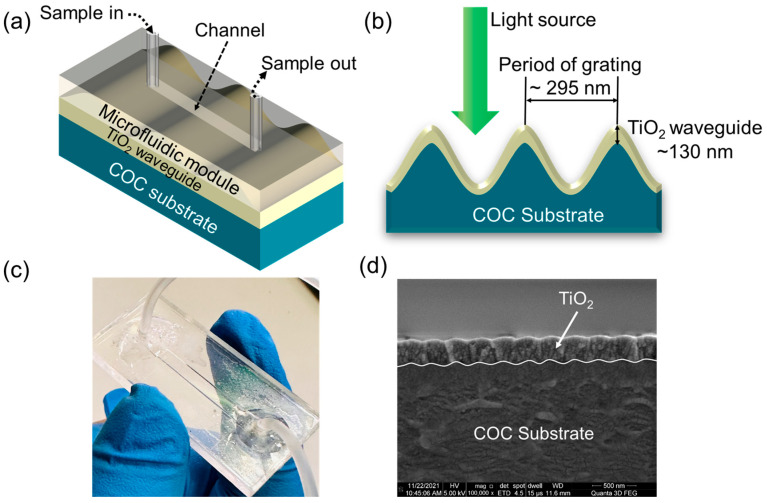
(**a**) The 3D and (**b**) 2D schematics of the GMR sensor chips utilized in this study. (**c**) The optical image and (**d**) the scanning electron microscopy (SEM) image of the fabricated sensor.

The three-dimensional (3D) and two-dimensional (2D) configurations of the GMR sensors are shown in Figure 1a and Figure 1b, respectively, whereas the optical and scanning electron microscopy (SEM) images of the fabricated sensor are shown in Figure 1c and Figure 1d, respectively.

**Figure 2:**
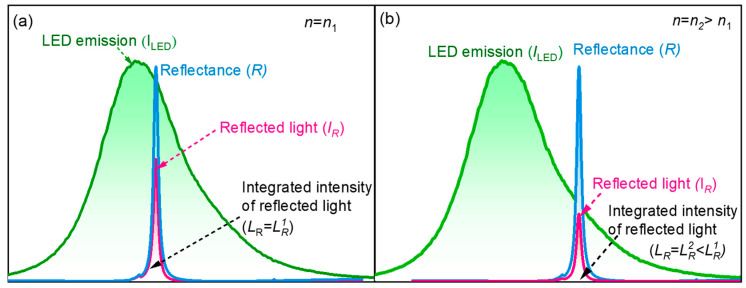
The working principle of the GMR sensor operating in the reflection mode for (**a**) *n* = *n*_1_ and (**b**) *n* = *n*_2_ > *n*_1_. (For the experimental green LED’s emission spectrum and reflectance spectrum of the proposed GMR sensor).

The GMR sensor operating in reflection mode works on the principle of the wavelength-dependent reflection induced by the excitation of leaky modes within a waveguide grating when illuminated with incident light. This resonance phenomenon occurs when light is incident onto the waveguide layer patterned with a grating and covered with a sample solution of RI (*n*) [2]. The interaction of the incident light with the periodic structure leads to the excitation of guided modes, resulting in sharp resonance peaks in the reflection spectrum, with a full-width at half-maximum (FWHM) of several nanometers depending on the quality factor of the grating structure, as shown in Figure 2. When operated in reflection mode, the sensor’s optical response is characterized by the reflectance spectrum, denoted as *R*(*n*, λ), which is highly sensitive to changes in the surrounding RI. As the incident light illuminates the sensor, a portion of the light is reflected and is denoted by *I*_R_. The integrated intensity of this reflected light, *L*_R_, depends on the overlap between the reflectance spectrum of the GMR structure and the spectral profile of the light source, which in our case is a green-light-emitting diode (LED) denoted by *L*_LED_(λ). *L*_LED_(λ) has a spectrally limited, Gaussian-like spectrum (denoted by *I*_LED_) with a wider FWHM of ∼30 nm, as shown in Figure 2a for RI *n*_1_. This relationship can be expressed as $L_{R}\left(n\right)=\int L_{LED}\left(\lambda \right)R\left(n,\lambda \right)d\lambda$. The LED spectrum is fixed, while the GMR reflectance spectrum varies with the RI of the analyte. When the RI increases to *n*_2_ (*n*_2_ > *n*_1_), it induces a redshift in the resonance peak of the reflectance spectrum, thereby leading to a decrease in the overlap between the GMR reflectance spectrum and the LED spectrum, as shown in Figure 2b. Hence, the integrated intensity of the reflected light decreases with the increase in the RI of the solution. This principle can be used to convert the spectrum drift signal into a reflected light intensity signal. Hence, the GMR sensor in reflection mode provides a powerful yet low-cost platform for label-free biosensing. By exploiting the precise spectral characteristics of GMR and a tailored spectrum caused by changes in the sample’s RI, the system can detect shifts in resonance. The integration of intensity-based detection not only simplifies the readout mechanism but also supports miniaturization and portability, making it particularly suitable for on-site and point-of-care applications.

## 3. Materials and Methods

### 3.1. Fabrication of Sensors

**Figure 3:**
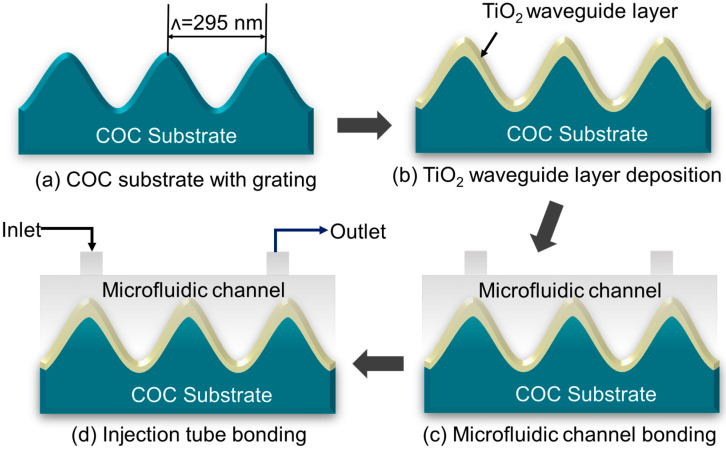
The fabrication flow of the GMR sensor.

The GMR biosensors were fabricated using a combination of injection molding and electron beam evaporator techniques, as shown in Figure 3. The fabrication process consists of three main steps: (i) preparation of cyclic olefin copolymer (COC) (ELITE PLASTIC INTERNATIONAL Co., Ltd., Taipei, Taiwan) substrate using injection molding technique, (ii) deposition of the TiO_2_ waveguide layer, and (iii) integration of the microfluidic module.

In the first step, a grating structure was fabricated on COC substrates using an injection molding technique [40]. A polydimethylsiloxane (PDMS) (Sil-More Industrial Ltd., Taipei, Taiwan) layer was transferred onto a stainless-steel mold core precoated with a sol–gel (Kelly Chemical Co., Nantou, Taiwan) solution using spin coating. The PDMS–mold assembly was baked, followed by UV curing to solidify the sol–gel structure. After curing, the PDMS layer was removed, and the stainless-steel mold was further baked to harden the sol–gel. The hardened mold was then used in the injection molding process to replicate the grating on COC substrates. The grating structure had a period of ~295 nm and a depth of ~100 nm optimized for GMR. A grating period of ~295 nm was selected for the GMR sensor design owing to its ability to achieve a resonance wavelength of ~520 nm, which is close to the resonance wavelength of green LED. Grating period of ~295 nm corresponds to a simulated FWHM of 1.77 nm. Although the sensitivity at this period is 62.1 nm/RIU, our primary focus was on aligning the resonance wavelength with the LED source. The selected grating period thus ensures both spectral alignment and a narrower FWHM, which aligns with our intended goal. To form the waveguide layer, a ~130 nm thick (measured using an AlphaStep surface profiler (SCIENTECH, Taipei, Taiwan)) TiO_2_ layer was deposited onto the injection-molded COC grating substrates using electron beam evaporation. A quartz crystal oscillator was utilized to monitor and control the deposition rate, ensuring uniform film thickness. The oscillator was replaced periodically to maintain accuracy during the deposition process. To facilitate liquid sample handling, the GMR sensor was integrated with a microfluidic module. A COC microfluidic cover, incorporating a sensing region, was aligned and bonded to the TiO_2_-coated COC substrate. UV adhesive (Everwide chemical, Yunlin, Taiwan) was applied between the microfluidic cover and the substrate and subsequently cured using UV light to ensure strong adhesion. Flexible plastic tubes (Yu Shun Instrument Co., Ltd., Chiayi, Taiwan) were attached to the inlet and outlet ports of the fluidic channel using AB adhesive (mixed in a 1:1 volume ratio) to secure the tubing connections. The resulting GMR sensor provides a low-cost, durable, and stable label-free sensing platform, offering reliable optical detection capabilities for biomolecular sensing applications.

### 3.2. Optical Detection System

The Jones matrix formalism was employed to achieve precise polarization control and streamline the operation of the optical detection system. This mathematical framework allows for systematic modeling of the polarization behavior of light as it propagates through a sequence of optical components. Each component within the system, such as polarizers, wave plates, and beam splitters, is represented by a corresponding 2 × 2 Jones matrix. By multiplying these matrices in the order that the light encounters each element, the overall transformation of the polarization state can be analytically determined. The resultant matrix provides a direct representation of the final polarization state of the output light. In particular, the polarization beam splitter (PBS) is modeled using Jones matrices that reflect its functionality: it transmits transverse magnetic (TM)-polarized light while reflecting transverse electric (TE)-polarized light. This dual behavior is incorporated into the system’s Jones model based on the directionality and orientation of incident light. The overall polarization response of the system can be computed using the sequential multiplication of individual Jones matrices, as detailed in Equations (1)–(6). By adopting this matrix-based modeling approach, manual adjustments of the polarization components are not required during sensing experiments. This significantly reduces system complexity, minimizes alignment errors, and enhances reproducibility and operational efficiency. It allows for the design of a more compact and user-friendly optical setup, particularly beneficial for real-time biosensing applications. Table 1 presents the Jones matrices for the primary optical components used in the system, offering a concise reference that correlates each element to its corresponding polarization transformation. This table not only facilitates a clearer understanding of the role of each component but also supports the design and simulation of advanced polarization-dependent optical systems [36,40,41].

**Figure 4:**
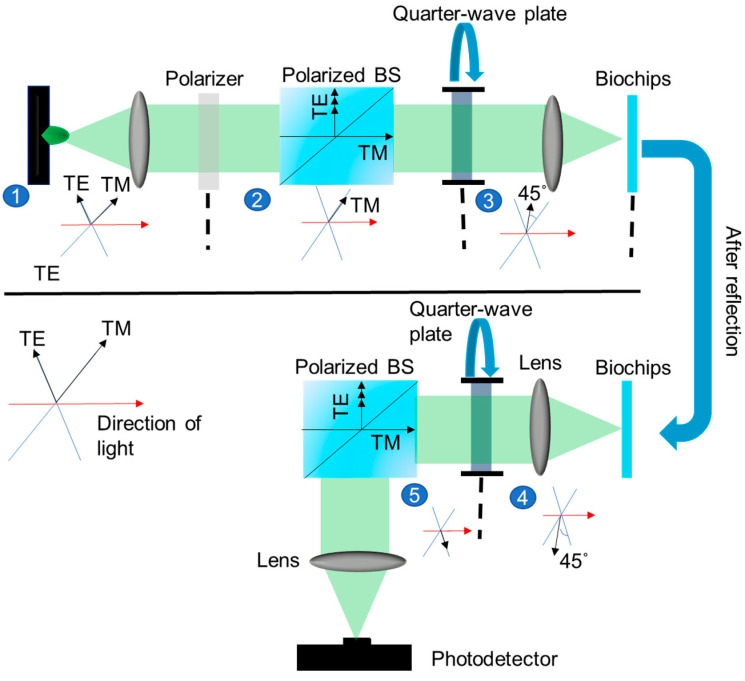
Designed optical detection system.

The proposed optical detection system (Figure 4) uses a low-cost and highly stable commercial green LED powered by signal generator as the light source. The TE mode of the collimated light is filtered via polarizer, and therefore, only the TM mode passes through the beam splitter and is further rotated 45° by the first 1/4 **λ** wave plate. The rotated mode is focused on the GMR sensor by another pair of lenses. The sensor reflected signal passes through the collimating lens following the 1/4 **λ** wave plate mode for the second time and rotates again. At this time, the TE mode enters the beam splitter, and finally, the light is focused on a photodetector (DET36A2, Onset Electro-Optics Co., Ltd., Taipei, Taiwan) through a focusing lens. This system successfully solves the overlapping problem of the signal and original light sources and filters out the background signal through the polarization beam splitter to improve the $S_{n}$ of the system. This mode is transformed through a series of optical elements as follows:

**Polarizer**: The light is filtered to permit only the TM-polarized component, represented by the Jones matrix:

(1) $$\left[\begin{array}{c} E_{x}\\ 0 \end{array}\right]=\left[\begin{array}{c} E_{x}\\ E_{y} \end{array}\right]\left[\begin{array}{cc} 1 & 0\\ 0 & 0 \end{array}\right]$$

**Polarizing Beam Splitter**: Following the polarizer, the light proceeds through the polarizing beam splitter, with the TM-polarized light passing through unchanged:

(2) $$\left[\begin{array}{c} E_{x}\\ 0 \end{array}\right]=\left[\begin{array}{c} E_{x}\\ 0 \end{array}\right]\left[\begin{array}{cc} 1 & 0\\ 0 & 0 \end{array}\right]$$

**Quarter-Wave Plate (45°)**: The remaining TM wave experiences circular polarization upon traversing the quarter-wave plate, represented as follows:

(3) $$\frac{1}{2}\left[\begin{array}{c} E_{x}\left(1-i\right)\\ E_{x}(1+i) \end{array}\right]=\frac{1}{2}\left[\begin{array}{c} E_{x}\\ 0 \end{array}\right]\left[\begin{array}{cc} 1-i & 1+i\\ 1+i & 1-i \end{array}\right]$$

**Chip Reflection**: The circularly polarized light is reflected through the chip, resulting in a sign change in the matrix multiplication, as shown in Equation (4):

(4) $$\frac{1}{2}\left[\begin{array}{c} E_{x}(1-i)\\ -E_{x}(1+i) \end{array}\right]=\frac{1}{2}\left[\begin{array}{c} E_{x}(1-i)\\ -E_{x}(1+i) \end{array}\right]\left[\begin{array}{cc} 1 & 0\\ 0 & -1 \end{array}\right]$$

**Quarter-Wave Plate (Reverse Direction)**: The reflected light interacts with the quarter-wave plate once again, resulting in

(5) $$\frac{1}{4}\left[\begin{array}{c} 0\\ -4E_{x} \end{array}\right]=\frac{1}{2}\left[\begin{array}{c} E_{x}(1-i)\\ -E_{x}(1+i) \end{array}\right]\frac{1}{2}\left[\begin{array}{cc} 1-i & -1-i\\ -1-i & 1-i \end{array}\right]$$

**Polarizing Beam Splitter (TE Mode)**: The light in a TE wave state traverses the final polarizing beam splitter, as follows:

(6) $$\left[\begin{array}{c} 0\\ -E_{x} \end{array}\right]=\frac{1}{4}\left[\begin{array}{c} 0\\ -4E_{x} \end{array}\right]\left[\begin{array}{c} 0\\ -E_{x} \end{array}\right]$$

These sequential transformations ensure the transition of the polarization state back to TE after reflection, allowing it to be directed to the photodetector. The Jones matrix calculations validate the consistent achievement of the desired polarization state, eliminating the need for manual adjustments. The designed optical detection system reduces the operational complexity and cost by eliminating the need for spectrometers to validate resonant wavelength positions. This makes the system user-friendly and accessible even to non-expert operators. A major advantage of this approach is its ability to effectively filter out background signals. The incorporation of polarization elements ensures the transmission or reflection of only the desired polarization mode, enhancing the signal-to-noise ratio and enabling more accurate measurements. Therefore, the proposed detection system, with its combination of cost-effectiveness, ease of use, and enhanced sensitivity, holds significant potential for scientific and industrial applications, such as biosensing and optical metrology, where accurate and reliable polarization control is crucial.

## 4. Results and Discussion

### 4.1. Simulation Results

Finite element method (FEM) simulations were conducted to optimize the structure parameters of the proposed GMR sensor. The simulations were performed using a unit cell representation, with periodic boundary conditions applied along the *x*-direction to assess the field distribution of the sensor. To ensure accurate wave propagation modeling, perfectly matched layers (PMLs) were implemented at the boundaries of the simulation domain to absorb the outgoing waves and prevent artificial reflections. A plane wave light source was positioned at the lower section of the unit cell, propagating along the *z*-direction and incident normally from the bottom of the COC substrate. A power monitor was placed at the upper part of the unit cell to record the output light intensity. The wavelength-dependent refractive indices of the COC and TiO_2_ layers used in the FEM simulations are given by [29]

(7) $$n(TiO_{2})=4.74-9.85\lambda +3.164\lambda^{2}-6.44\lambda^{3}$$

(8) $$n(\mathrm{COC})=1.68-0.58\lambda +0.75\lambda^{2}-0.34\lambda^{3}$$

where λ is the free-space wavelength in micrometers (μm) . The grid size was set to λ/5. The grating period and grating height were varied from 266 nm to 466 nm and 50 to 200 nm, respectively. The TiO_2_ thickness was varied from 30 to 150 nm.

The grating period is a key parameter in the GMR sensing system, affecting not only the resonance wavelength but also the sensitivity and other characteristics of the GMR structure. The sensitivity of the sensor is determined by dividing the changes in the resonance wavelength ($∆\lambda_{R})$ and RI (∆ *n*), as follows [29]:

(9) $$S_{n}=\frac{\Delta \lambda_{R}}{\Delta n}$$

**Figure 5:**
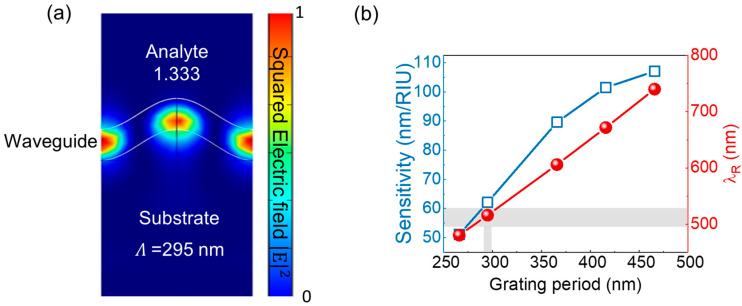
(**a**) The energy distribution of the proposed GMR structure for a grating period of 295 nm. (**b**) The sensitivity and resonant wavelength of the GMR structure as a function of the grating period.

The energy distribution within the GMR structure (Figure 5a) reveals a significant interaction between evanescent waves and the solutions under the test, owing to the complete GMR effect caused by the sufficient waveguide thickness. Figure 5b shows the relationship between the GMR structure’s grating period, sensitivity, and *λ*_R_ shifts. As the grating period increases from 266 nm to 466 nm, the sensitivity improves by 52.3%, reaching 107.1 nm/RIU, underscoring the strong dependence of the GMR performance on the grating period. Among the simulated grating periods, 295 nm was selected for the GMR sensor design, owing to its ability to achieve a *λ*_R_ of ~520 nm, which is closest to the resonance wavelength of the green LED light source.

**Figure 6:**
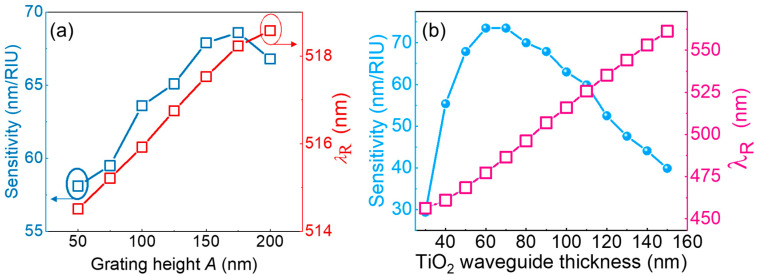
(**a**) Relationship between grating height, sensitivity, and resonant wavelength position. (**b**) Relationship between thickness of TiO_2_ waveguide, sensitivity, and resonant wavelength position.

The GMR sensor used in this study was fabricated using a nanoimprint technique, where variations in the imprint strength and master mold configuration can lead to changes in the grating height. To evaluate the impact of the grating height on the sensor performance, simulations were conducted for heights ranging from 50 nm to 200 nm while maintaining a fixed grating period of 295 nm. Figure 6a shows the relationship between the grating height, sensitivity, and λ_R_ shift. The results show that the sensitivity of the GMR structure increases with the grating height, reaching its peak at a height of 175 nm, which is 15.3% higher than that at 50 nm. The grating height minimally impacts the λ_R_ shift, as evidenced by the 4 nm increase observed across the height range from 50 nm to 200 nm. These findings indicate that variations in the grating height significantly affect the interaction of the evanescent wave with the analyte, thereby influencing sensitivity. Figure 6b illustrates the correlation between the TiO_2_ waveguide layer thickness, spectral sensitivity, and λ_R_ shift within a simulated GMR structure characterized by a grating period of 295 nm. The results show that as the thickness of the waveguide layer increases from 50 nm to 150 nm, the sensitivity peaks at 70 nm/RIU at 60 nm and subsequently decreases. The sensitivity decreases to 39.9 nm/RIU at 150 nm, representing a 45.3% reduction compared with the peak sensitivity. Below 60 nm, the sensitivity drops sharply, likely due to an incomplete GMR effect caused by an insufficient waveguide thickness. As the waveguide layer thickness increases, the *λ*_R_ shifts linearly from 456 nm to 561 nm, reaching 544 nm at a waveguide thickness of 130 nm, which aligns closely to the resonance wavelength of the green LED light source.

### 4.2. RI Sensing

Experiments were conducted to evaluate the RI sensing capabilities of the optofluidic GMR sensor. The experiment was conducted on five separate chips to ensure the reliability and repeatability of the results. Solutions were prepared with varying sucrose concentrations, each with a unique RI (n=1.333−1.373). Initially, a blank solution of deionized (DI) water (n=1.333) was injected into the GMR chip. A series of sucrose solutions with varying RIs were then introduced, and, finally, DI water was injected. A data acquisition system was utilized to monitor the variation in the light intensity. The normalized sensitivity $\left(S_{n}\right)$ and sensor resolution $\left(R_{s}\right)$, which signify the minimal detectable alteration in the RI of the solution, are defined using Equations (10) and (11), respectively:

(10) $$S_{n}=\frac{d}{dn}\left[\frac{I_{\mathrm{avg}}(n)}{I_{0}}\right]$$

(11) $$R_{s}=\frac{\sigma }{S}$$

where σ is the system noise governed by the baseline fluctuation in the output light intensity of the DI water, $I_{\mathrm{avg}}$ is the average intensity of the output light, and $I_{0}$ is the average light intensity measured from the DI water solution.

**Figure 7:**
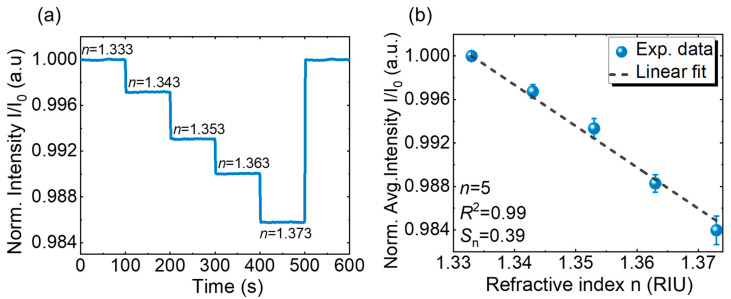
(**a**) RI sensing using the proposed detection system and (**b**) linear fitting for *n* = 5.

To assess the sensing performance of the fabricated GMR sensor, RI sensing experiments were conducted using the designed optical detection system. The system’s response to changes in the RI was assessed by sequentially injecting sucrose solutions of varying concentrations into the chip. The real-time RI sensing outcomes for the sensor with a grating period of ~295 nm are shown in Figure 7a. The intensity of the output light decreases with the increasing RI, forming a step-like profile. This behavior arises because the resonant wavelength becomes closer to the resonance wavelength of the green LED light source, which lies on the right side of the emission peak of the green LED used in this study. As the RI increases, the resonant wavelength shifts toward longer wavelengths, moving further away from the LED emission peak and resulting in a corresponding decrease in the output intensity. The real-time sensing data for n=1.333 demonstrate an exceptional power stability of σ=0.00326%. This performance significantly exceeds the previously reported σ=0.01% in GMR optofluidic biosensors that utilize highly stable laser sources [33]. This exceptional stability is an indication of low noise levels in our system, attributed to the LED’s high-power consistency and the lock-in detection method, which effectively enhance the signal-to-noise ratio. By utilizing the measured values of the sensitivity (S) and power stability (σ), the RI resolution of the system was determined to be $R_{s}=8.15\times 10^{-5}\;\mathrm{RIU}$. An increase in the sample’s RI increases the change in the light intensity. This behavior is attributed to a GMR wavelength shift, which alters the spectral overlap between the LED emission and the sensor’s spectrum.

The calibration curve of the real-time optical responses of the proposed sensor in relation to the RI of the sample solution is shown in Figure 7b. By applying Equations (10) and (11), a linear fit of the data provides a normalized sensitivity of $S_{n}=0.4\;\mathrm{RI}U^{-1}$, with a linear correlation coefficient of $R^{2}=0.993$. The obtained calibration curve exhibited a clear and consistent response to RI changes despite the polarization angle remaining fixed throughout the measurement process. This confirms that the Jones matrix-based optical system design ensures a stable and accurate GMR excitation, enabling a precise and repeatable RI detection while significantly simplifying the system operation.

Table 2 compares the fabrication method, resolution, and cost performance of the proposed GMR sensor with other GMR-based and optical sensors reported in the literature. The proposed sensor demonstrates a unique combination of a high resolution and low cost, which is not concurrently achievable by the other sensors. Although some platforms offer high resolutions, they often require expensive instrumentation or complex alignment procedures. By contrast, low-cost designs frequently compromise on the sensitivity or measurement stability. By integrating the Jones matrix-based analysis with a simplified low-cost optical setup, the proposed GMR sensor delivers a high resolution, placing it in a favorable position compared with existing technologies.

## 5. Conclusions

This study developed a GMR sensor for a swift and accurate RI detection, aiming to overcome the limitations of conventional GMR systems. The proposed sensor, together with an optimized optical detection system, simplifies the polarization state control, streamlines system operations, and resolves challenges related to overlapping signal light sources and optical signals. A high sensitivity was achieved by optimizing the structure parameters of the GMR sensor via FEM simulations. The proposed system enables rapid and accurate RI sensing with an RI resolution of $R_{s}=8.15\times 10^{-5}\;\mathrm{RIU}$ and a normalized sensitivity of $S_{n}=0.4\;\mathrm{RI}U^{-1}$. These advancements establish the proposed GMR platform as a user-friendly, cost-effective solution with an enhanced performance, making it well-suited for scientific and industrial applications, such as biosensing and optical metrology, where precise polarization control is critical.

## Figures and Tables

**Table 1:** The comparison of the Jones moment vibration across various components of the proposed optical detection system.

Component Type	Representation of the Jones Moment Oscillation of the Component
Green light source	$\left[\begin{array}{c} E_{x}\\ E_{y} \end{array}\right]$
Polarizer	$\left[\begin{array}{cc} 1 & 0\\ 0 & 0 \end{array}\right]$
Polarizing beam splitter TM	$\left[\begin{array}{cc} 1 & 0\\ 0 & 0 \end{array}\right]$
Quarter—wave plate 45°	$\frac{1}{2}\left[\begin{array}{cc} 1-i & 1+i\\ 1+i & 1-i \end{array}\right]$
Chip reflection	$\left[\begin{array}{cc} 1 & 0\\ 0 & -1 \end{array}\right]$
1/4 Wave plate (quarter-wave plate-45°) reverse	$\frac{1}{2}\left[\begin{array}{cc} 1-i & -1-i\\ -1-i & 1-i \end{array}\right]$
Polarizing beam splitter TE	$\left[\begin{array}{cc} 0 & 0\\ 0 & 1 \end{array}\right]$

**Table 2:** Performance parameters of the proposed GMR sensor with previously reported GMR and other sensor types.

Sensor Type	Fabrication Method	Resolution (RIU)	Cost	Ref.
Gold-coated SPR sensor	Contact lithography and nanoimprinting	$5.3\times 10^{-3}$	Low	[42]
PhC	PECVD	$1\times 10^{-3}$	Low	[43]
Bent waveguide	Spin coating and UV lithography	$5.31\times 10^{-4}$	Low	[44]
Functional polymer-based planar optical waveguide	Spin coating	$4.92\times 10^{-4}$	High	[45]
GMR sensor	Laser interference lithography	$1.62\times 10^{-4}$	Low	[46]
Interferometer	Soft lithography	$1.24\times 10^{-4}$	Low	[47]
Si prism and grating coupler	EB lithography	$8.8\times 10^{-5}$	High	[48]
GMR sensor	Injection molding and nanoimprinting.	$8.15\times 10^{-5}$	Low	This work

## Data Availability

Data supporting the findings of this study are available upon request from the corresponding authors. The data are not publicly available owing to privacy and ethical restrictions.
